# Novel ocular observations in a child with Joubert syndrome type 6 due to pathogenic variant in *TMEM67* gene

**DOI:** 10.1016/j.ajoc.2024.102091

**Published:** 2024-06-14

**Authors:** Maram EA Abdalla Elsayed, Syed M. Ali, Carly Gardner, Igor Kozak

**Affiliations:** a Oxford Eye Hospital, Oxford, UK; b Moorfields Eye Hospitals UAE, Abu Dhabi, United Arab Emirates; c Danat Al Emarat Hospital, Abu Dhabi, United Arab Emirates; d Mohammed Bin Rashed University, Dubai, United Arab Emirates; e Department of Diagnostic Radiology, Cleveland Clinic, Cleveland, OH, USA

**Keywords:** Joubert syndrome type 6, *TMEM67* gene, Molar tooth sign, Batwing sign, Subretinal fibrosis, Epipapillary fibrosis, Retinal detachment

## Abstract

**Purpose:**

To describe unique ocular features in a child with Joubert syndrome type 6.

**Observations:**

A 4-year-old male patient presented with right microphthalmia and non-dilating pupil and left primary position nystagmus. Brain MRI revealed a “molar tooth sign” of the midbrain and a “batwing sign” of the fourth ventricle along with large retroorbital cysts bilaterally. The diagnosis of autosomal recessive Joubert syndrome type 6 due to homozygous pathogenic variant c.725A > G p. (Asn242Ser) in *TMEM67* gene was confirmed by whole exome sequencing. Left eye had nystagmus and the left optic nerve and retina showed epipapillary and subretinal fibrosis, respectively. Scleral buckle was performed for left non-rhegmatogenous retinal detachment which then improved and has been stable.

**Conclusions and Importance:**

We present a rare case of JS with some unique ophthalmic features which expand clinical knowledge on this complex systemic and ocular entity.

## Highlights

• Joubert syndrome is a ciliopathy diagnosed via genetic testing or pathognomonic brain MRI findings.
• It can present numerous ocular features including oculomotor apraxia and retinal pathology mostly pigmentary retinopathy.
• Epipapillary and subretinal fibrosis can be present in Joubert syndrome.

## Teaser

After conducting a literature review on March 20, 2024 utilizing PubMed, Google Scholar and Web of Science search engines using the key words Joubert syndrome (review), eye, retina, optic nerve, fibrosis, we did not find any prior reports of epipapillary and/or subretinal fibrosis reported in any type of Joubert syndrome spectrum.

## 1 Introduction

Joubert syndrome (JS, MIM PS213300) is a ciliopathy which is characterized by malformation in the cerebellum and brainstem, recognizable on axial brain magnetic resonance imaging (MRI) as a “molar tooth sign” (MTS). Its characteristic clinical features include the triad of hypotonia, ataxia, and neurodevelopmental manifestations.^1^, ^2^, ^3^ Distinctive cerebellar and brainstem features consist of cerebellar vermis hypoplasia, deepened interpeduncular fossa, and thickened and elongated superior cerebellar peduncles. Additionally, the term Joubert syndrome-related disorders (JSRD) has been adopted to describe distinct neuroradiological feature of MTS while involving other organ systems. Based on organ involvement, JSRD is classified into phenotypes: JS with renal defect, JS with ocular defect (pure JS), JS with oculorenal defects, JS with hepatic defects, and JS with orofaciodigital defects.^4^

Joubert syndrome is a very rare genetic disease with a prevalence of 1:55.000–1:200.000.^5^ It demonstrates significant genetic heterogeneity that exhibits predominately autosomal recessive, and rarely X-linked inheritance. Being a ciliopathy, clinical features involve mainly the retina and the kidney. Ocular and oculomotor phenotypes include nystagmus (typically see-saw), strabismus, oculomotor apraxia, vertical gaze palsy, congenital retinal dystrophy, pigmentary retinopathy, ocular coloboma and ptosis.^6^ More than 40 genes are known to cause the disease, including the *AHI1* (chromosome 6) and *CEP290* (chromosome 13) genes critical in the primary cilia function.^6^ Some genetic causes (e.g., CEP290 and AHI1 dysfunction) are more likely to be associated with more severe retinal degeneration than others (e.g., INPP5E, MKS1, and NPHP1 dysfunction).^7^

Herein we present a rare case of genetically confirmed autosomal recessive JS with some unique ophthalmic features and report on their characteristics and follow-up observation.

## 2 Case report

A 4-year old male patient from a consanguineous family from Bahrain, initially diagnosed with Joubert syndrome by pediatric neurology and later confirmed by genetic testing, was referred for the second opinion and management of a retinal detachment. He was the product of an unremarkable pregnancy and full term spontaneous vaginal delivery. The child suffered from overall hypotonia, difficulty walking and severe neurodevelopmental deficit. External examination revealed bilateral small eyes with right microphthalmia and left primary position nystagmus. Due to poor cooperation, examination under anesthesia demonstrated hyperopic astigmatism and intraocular pressures of 2.7 mmHg and 2.9 mmHg in the right and left eyes, respectively. There was bilateral microphthalmos and microcornea (corneal diameter OD = 7 mm, OS = 9.5 mm). Right anterior segment examination showed white dots on the iris surface, non-dilating pupil and lenticular membranes precluding view to retina. B-scan showed right microphthalmos with a retrobulbar cyst.

**Fig. 1:**
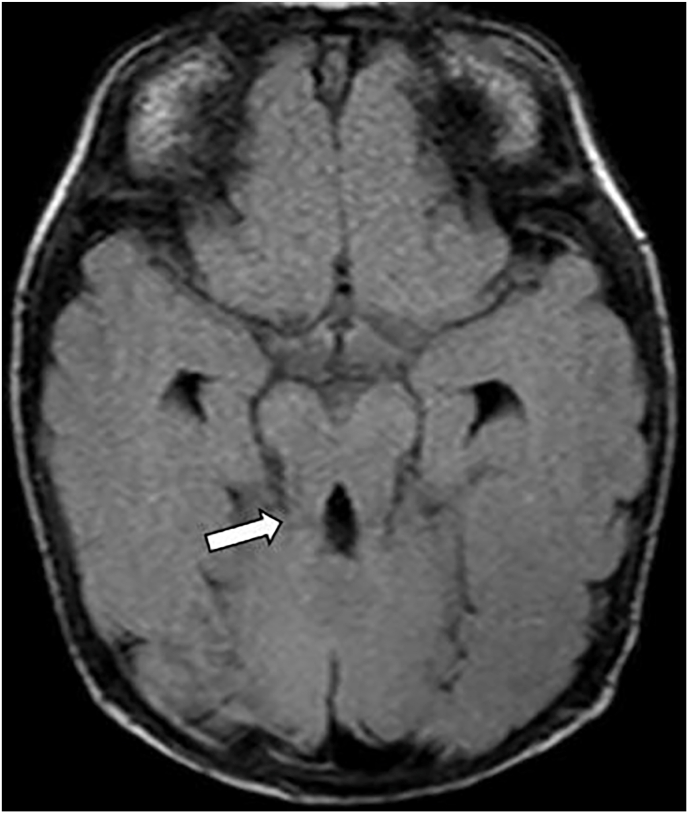
Axial T1 weighted magnetic resonance image (MRI) shows the classic “molar tooth sign” of the midbrain-hindbrain (white arrow) pathognomonic for Joubert syndrome.

**Fig. 2:**
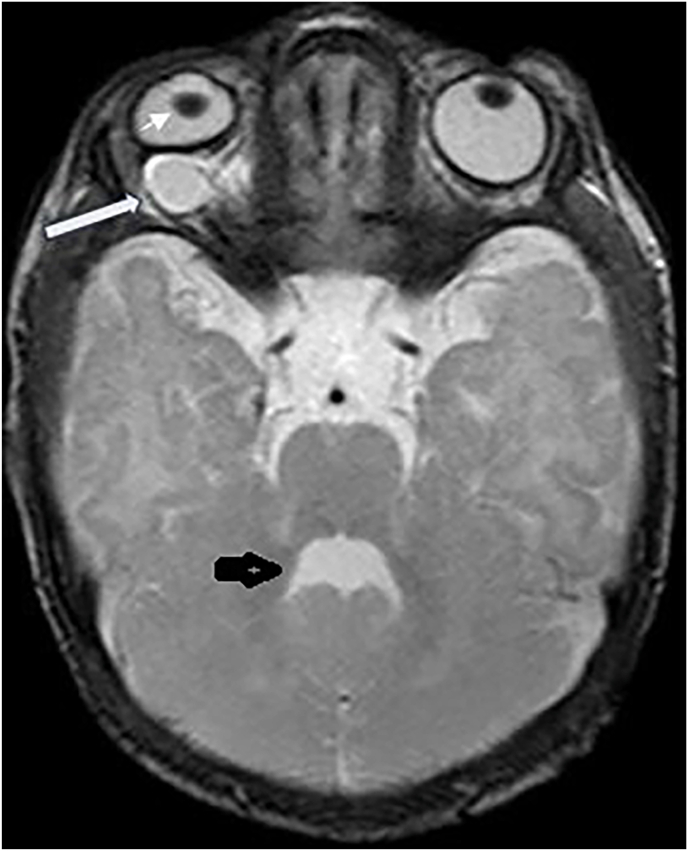
Axial T2 weighted MR image shows right retrobulbar cysts (long white arrow) with deformity of the globe and right microphthalmia and lens subluxation. Fourth ventricle shows “batwing or umbrella sign” characteristic of Joubert syndrome (short black arrow).

**Fig. 3:**
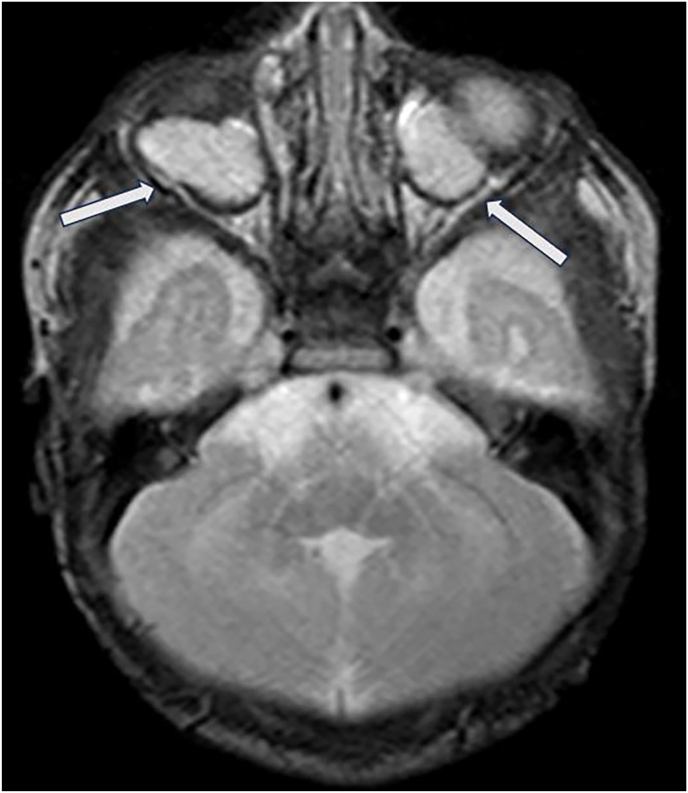
Axial T2 weighted MR image shows retrobulbar cysts (white arrows) with deformity of the globes bilaterally and right microphthalmia.

**Fig. 4:**
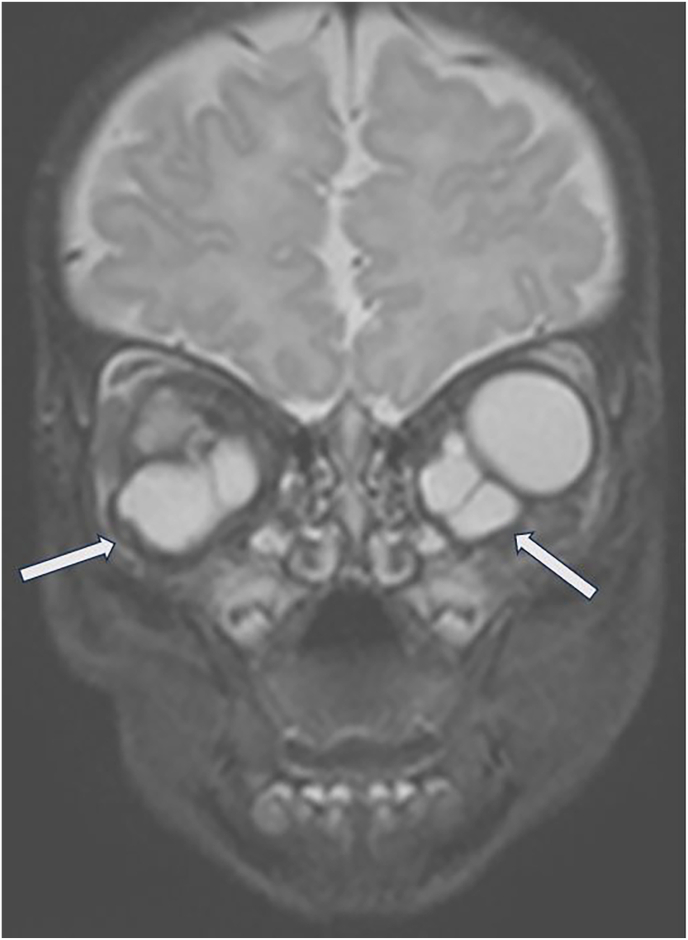
Coronal T2 weighted MR image of the orbits shows bilateral multiloculated retrobulbar cysts (white arrows).

Prior brain imaging demonstrated the classic “molar tooth sign” of the midbrain-hindbrain which is pathognomonic for Joubert syndrome (Fig. 1) and “batwing sign” of the fourth ventricle characteristic of Joubert syndrome (Fig. 2). Additionally, MRI scans have shown bilateral microphthalmia, retrobulbar cysts with deformity of the globes bilaterally and right lens subluxation (Fig. 3, Fig. 4). The whole exome sequencing (Illumina platform) detected a homozygous pathogenic variant c.725A > G p. (Asn242Ser) in *TMEM67* gene. It is classified as class 1 (pathogenic) according to the recommendations of ACMG and CENTOGENE® (Clinical Laboratory Improvement Amendments (CLIA) certified analyzing laboratory). An incidental finding was a heterozygous pathogenic class 1 variant in the *PKP2* gene which increases genetic susceptibility to the autosomal dominant arrhytmogenic right ventricular dysplasia 9.

**Fig. 5:**
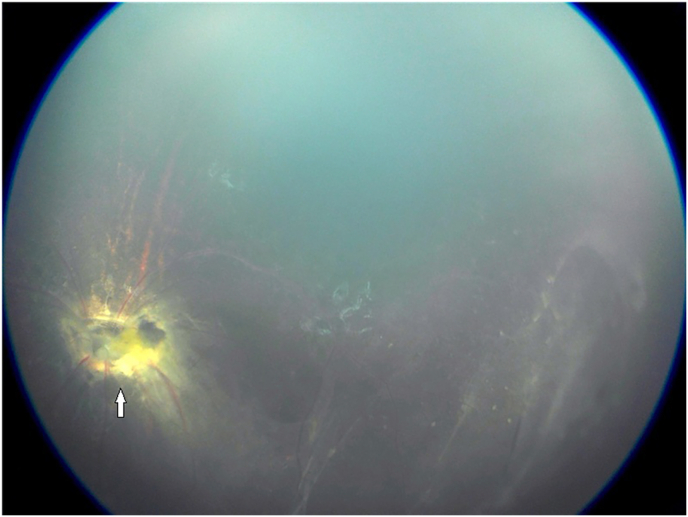
Wide-field color fundus photography shows a dysplastic optic nerve with epipapillary fibrosis (white arrow) and some subretinal fibrosis inferiorly and temporally.

**Fig. 6:**
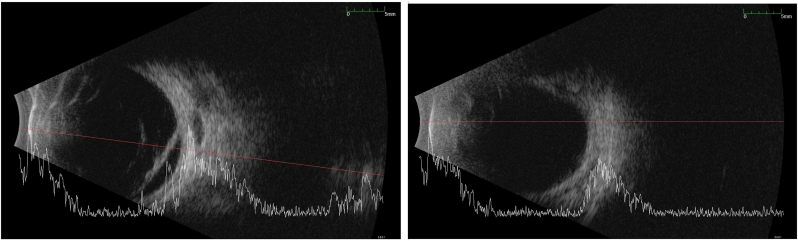
Left Panel: Pre-operative ultrasound image demonstrates retinal detachment. Right Panel: Ultrasound image following scleral buckle surgery of the same eye shows slightly elongated eye with attached retina.

**Fig. 7:**
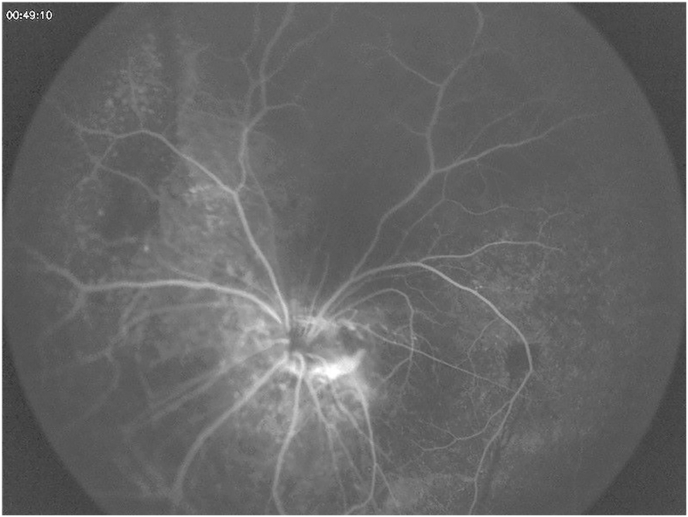
Wide-field color fundus fluorescein angiography in early frames demonstrates good retinal perfusion with mild staining of the retinal pigment epithelium.

**Fig. 8:**
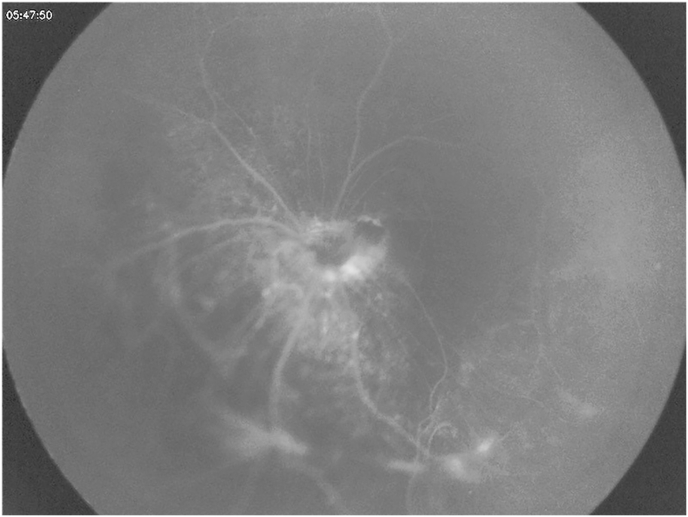
Wide-field color fundus fluorescein angiography in late frames demonstrates mild leakage at the optic nerve margin and retinal vessels most likely due to vascular distortion and dysplasia. No active neovascularization was seen.

Dilated left fundal exam showed a dysplastic optic nerve with epipapillary fibrosis (Fig. 5). Although the superior retina was attached, there were areas of subretinal fibrosis and retinal detachment inferiorly and temporally with severe distortion of the retinal vessels. There was no retinal exudate and no breaks were visible. There was no epiretinal membrane but mild subretinal fluid confirmed by B-scan (Fig. 6). Fundus fluorescein angiography revealed inferior retinal neovascularization with leakage in late frames and absent retinal vasculature temporally - maldevelopment consistent with syndromic features (Fig. 7, Fig. 8). After extensive discussion with the parents regarding guarded visual prognosis, we proceeded with extraocular scleral buckling to relieve some ongoing traction of the inferior retina. A 360° conjunctival peritomy was performed followed by placing the extraocular muscles on bridal sutures, and the placement of a silicone band tied with #90 sleeve. The band was secured by sutures in all 4 quadrants and its tightness was adjusted. Tenons and conjunctiva were apposed and sutured using 8.0-Vicryl sutures. Subconjunctival antibiotics and steroids were administered followed by standard post-operative outpatient care.

**Fig. 9:**
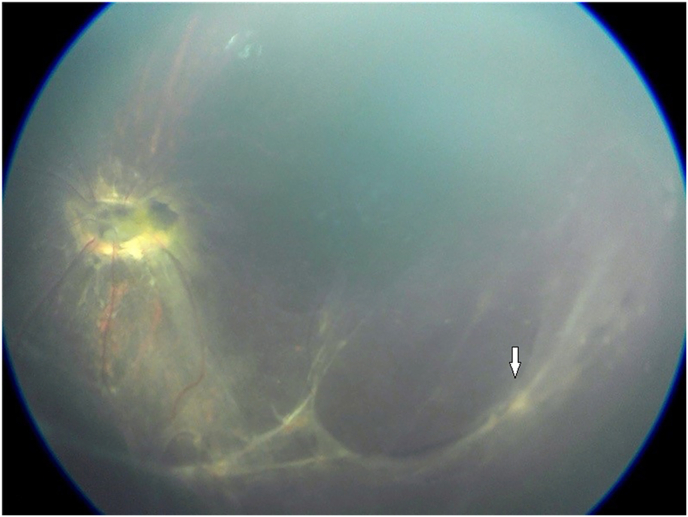
Wide-field color fundus photography after scleral buckling surgery shows a dysplastic optic nerve with epipapillary fibrosis and a mild increase in subretinal fibrosis with no change in retinal attachment (white arrow).

One month after surgery the left retina remained stable post scleral buckle surgery with inactive subretinal fibrosis and no fluid (Fig. 6). Examination under anesthesia 3 months after surgery showed stable attached retina. At 6 months post-op there was a mild increase in subretinal fibrosis (Fig. 9) with attached retina and hence no further interventions were necessary. The child undergoes regular visits with cycloplegic refraction. Subsequent exams have shown stable ocular and retinal findings up to 3 years following surgery.

## 3 Discussion

While our case highlights a wide array of systemic and ocular pathology in JS, some features presented in our patient are quite unique. This is a monocular patient who presented with epipapillary and subretinal fibrosis and localized retinal detachment with potential to expand. The extraocular surgery stabilized the condition and is probably a preferred approach in contrast to more invasive intraocular vitrectomy with drainage and tamponade. Given unknown nature of subretinal fibrosis, entry to subretinal space could potentially risk triggering of unwanted proliferative changes. The retina had pigmentary changes and dysplastic features. While pigmentary retinopathy including retinal dystrophy is the most common reported ocular pathology in JS,^6^ after conducting a literature review on December 2, 2023 utilizing PubMed, Google Scholar and Web of Science search engines using the key words Joubert syndrome (review), eye, retina, optic nerve, fibrosis, we did not find any prior reports of epipapillary and/or subretinal fibrosis reported in Joubert syndrome. Other rare retinal finding in this case include retinal dysplasia with leakage on fluorescein angiogram without neovascularization. This retinal dysplasia included retinal vessel distortion which could be the reason for fluorescein dye leakage due to weakening of blood-retina barrier. Similarly in optic nerve pathology in JS, the literature reports on Leber congenital amaurosis, optic nerve drusen and coloboma^6^^,^^8^ but epipapillary fibrosis is yet to be reported. This fibrotic tissue showed mild fluorescein leakage at the optic nerve margin in late frames on fluorescein angiogram.

A rare findings in our patient is the presence of bilateral microphthalmia with ocular hypotonia and multiple retrobulbar cysts. Only three prior JS cases in the literature report the presence of retrobulbar cysts.^6^^,^^9^ They were large and thin-walled. The cysts in this patient were multilobulated, large, distorting right globe and perhaps contributing to arrest of its development. Other reported orbital lesion in Joubert syndrome due to a pathogenic variant in the same *TMEM67* gene includes a case of bilateral opticmeningoceles.^10^ While intraocular examination was not possible clinically, MRI revealed intraocular lens subluxation in the right, microphthalmic eye. Dilation was possible in the left eye in which intraocular lens was in situ. The findings of strabismus and nystagmus, present in this child, are common in JS.^6^

JS is part of the larger group of ciliopathies which are based on shared genetic causes and overlapping phenotypes related to dysfunction of the primary cilium – a modified cilium connecting the inner and outer segments of photoreceptors. Maintenance of photoreceptor integrity relies on continuous intraflagellar transport.^11^ Our patient had pathogenic variants in the transmembrane protein 67 (*TMEM67*) gene which plays a role in ciliogenesis and pathogenic variants in this gene cause >10 % of JS cases.^12^, ^13^, ^14^, ^15^, ^16^ This variant causes mostly autosomal recessive pattern of inheritance (OMIM 609884).^17^, ^18^, ^19^ The same variants have been previously described with associated abnormal eye movements but without ocular findings reported here.^20^, ^21^, ^22^ A second pathogenic variant in the protein Plakophilin-2 (*PKP2*) gene predisposing arrhytmogenic right ventricular dysplasia 9 was an accidental finding and the patient did not have cardiac problems. Defects in *PKP2* gene *in vitro* increase expression of profibrotic genes which is a plausible connection to increased pre- and subretinal fibrosis in this case.^23^ Other common disorder in ciliopathies is kidney dysfunction which was not present in this case. The parents were informed about guarded visual prognosis and a careful vigilance for both new ocular and systemic symptoms and signs.

## 4 Conclusions

We present a complex syndromic case of genetically confirmed autosomal recessive JS with the presence of two novel ocular features (subretinal and epipapillary fibrosis) expanding its phenotypic spectrum. We also describe the management of the non-rhegmatogenous retinal detachment observed in this case. The significance of early detection of the syndrome is stressed so that clinicians can promptly assess for visual impairment and provide available treatment(s) and supportive measures to individuals and their families. Ideally, a baseline assessment for the issues described above would be performed when JS is diagnosed or suspected, with regular ophthalmological reviews for surveillance and treatment of possible retinal developments such as retinal detachments.

## Patient consent

The parents have given consent to publish this case.

## Funding

No funding or grant support.

## Authorship

All authors attest that they meet the current ICMJE criteria for Authorship.

## CRediT authorship contribution statement

**Maram EA Abdalla Elsayed:** Writing – original draft. **Syed M. Ali:** Writing – review & editing, Conceptualization. **Carly Gardner:** Writing – review & editing. **Igor Kozak:** Writing – review & editing, Conceptualization.

## Declaration of competing interest

The authors declare that they have no known competing financial interests or personal relationships that could have appeared to influence the work reported in this paper.
